# Knowledge, Attitudes, and Practices (KAP) Regarding the COVID-19 Outbreak in Côte d’Ivoire: Understanding the Non-Compliance of Populations with Non-Pharmaceutical Interventions

**DOI:** 10.3390/ijerph18094757

**Published:** 2021-04-29

**Authors:** Richard B. Yapi, Clarisse A. Houngbedji, Daniel K.G. N’Guessan, Arlette O. Dindé, Aimé R. Sanhoun, Ariane Amin, Kossia D.T. Gboko, Kathrin Heitz-Tokpa, Gilbert Fokou, Bassirou Bonfoh

**Affiliations:** 1 Centre d’Entomologie Médicale et Vétérinaire, Université Alassane Ouattara, Bouaké 01 BPV 18, Côte d’Ivoire; Clarisse.houngbedji@csrs.ci; 2 Centre Suisse de Recherches Scientifiques en Côte d’Ivoire, Abidjan 01 BP 1303, Côte d’Ivoire; k_daniel00@yahoo.fr (D.K.G.N.); arlette.dinde@csrs.ci (A.O.D.); aime.sanhoun@csrs.ci (A.R.S.); ariane.amin@csrs.ci (A.A.); kossia.gboko@csrs.ci (K.D.T.G.); kathrin.heitz-tokpa@csrs.ci (K.H.-T.); gilbert.fokou@csrs.ci (G.F.); bassirou.bonfoh@csrs.ci (B.B.); 3 UFR des Sciences et Technologies des Aliments, Université Nangui Abrogoua, Abidjan 02 BP 801, Côte d’Ivoire; 4 UFR des Sciences Economiques et de Gestion, Université Felix Houphouët Boigny, Abidjan 01 BPV 34, Côte d’Ivoire; 5 Institute of Social Anthropology, University of Basel, Petersplatz 1, 4001 Basel, Switzerland; 6 Developmental, Capable and Ethical State (DCES) Division, Human Sciences Research Council (HSRC), 116-118 Buitengracht Street, Private Bag X9182, Cape Town 8000, South Africa

**Keywords:** COVID-19, Côte d’Ivoire, compliance, knowledge, attitudes, practices

## Abstract

At the beginning of the COVID-19 outbreak, preventive measures seemed the most appropriate method to control its spread. We assessed the knowledge, attitudes, and practices of the Ivorian public regarding preventive measures, conducting a hybrid survey across the country. Participants were invited to complete a questionnaire online, by phone, or face-to-face. Chi-squared, Fisher’s exact, and Kruskal–Wallis tests were used to compare the frequency of responses regarding compliance with preventive measures. Data were validated for 564 individuals. Over one-third of respondents believed that COVID-19 was related to non-natural causes. Though the disease was perceived as severe, respondents did not consider it to be highly infectious. Overall, 35.6% of respondents fully trust health officials in the management of the pandemic, and 34.6% trusted them moderately. Individuals who believed COVID-19 was a disease caused by a pathogen and the well-educated were likely to comply with preventive measures. About 70% of respondents stated that their daily expenses had increased due to preventive measures. The study concludes that beyond unfavorable socioeconomic conditions, the level of knowledge regarding COVID-19 and trust in the government/health system are more likely to influence compliance with preventive measures such as self-reporting, physical distancing, the use of face masks, and eventually the acceptability of vaccines.

## 1. Introduction

The novel severe acute respiratory syndrome coronavirus (SARS-CoV-2) that causes COVID-19 was first reported in December 2019 in Wuhan, Hubei province, China. The disease rapidly spread throughout the world, becoming a public health emergency of international concern a month later [1]. This situation has disrupted social and community life and affected all types of human activities, severely disturbing national and international travel, supply chains, and the global manufacturing industry. In March 2020, the disease was declared a pandemic by the World Health Organization (WHO) [2], which called for all countries to come together to take concerted action and implement strategies to combat the threat caused by this virus. In Africa, the first case of the disease was officially recorded on 14 February 2020 in Egypt, and shortly after, new cases were reported everywhere on the continent, with the spread driven mainly by international air travel [3].

A wholesale wet market in Wuhan, China, has been identified as a possible source of the initial SARS-CoV-2 outbreak. Pangolins were identified as the likely intermediate host of the virus due to sequence similarities between SARS-CoV-2 and a SARS-CoV-like virus identified in pangolin lung samples. Thus, SARS-CoV-2 is considered a zoonotic infection [4]. After the presumed initial animal–human infection, transmission now mainly occurs through human–human contact. The disease is highly contagious, and transmission depends on the frequency and duration of contact between people. At the beginning of the pandemic, the reproductive number (*R*_0_) was around 3.28. [5]. In the absence of efficient control measures, the number of infected people in need of care can grow faster in few days, which can put a strain on any national health care system, even robust and well-supplied ones.

African countries were amongst the latest to be affected by the disease. The spread of the novel SARS-CoV-2 virus in this part of the world was worrisome because of the poor organization and lack of resources within health systems, co-morbidities amongst the population, and specific living conditions (i.e., population density in urban areas and lack of access to water, hygienic conditions, and sanitation). In the absence of an effective vaccine and medication against COVID-19, various governments around the world have resorted to drastic measures to slow down the transmission to reduce pressure on healthcare providers. Most African countries adopted a ‘suppression strategy’ aiming to avoid transmission and keep cases to an absolute minimum for as long as possible through lockdowns, quarantine regulations, contact tracing, and the closure of businesses, schools, and universities [6]. These restrictions were accompanied by preventive measures such as social distancing in all age groups, handwashing, and face mask-wearing. In comparison to vaccines and medications, preventive measures (classed as non-pharmaceutical interventions, NPIs), are a cost-effective option to fight the spread of COVID-19. The compliance of the population with preventive measures is thus a crucial component in disease control strategies in low-income countries which face financial constraints for public health in general [7]. However, community buy-in to control measures may be largely affected by people’s knowledge, attitudes, and practices (KAP) towards COVID-19. Lessons learnt from the SARS outbreak in 2003 suggest that knowledge and attitudes towards infectious diseases are associated with the level of panic and emotion in the population, which can further complicate attempts to prevent the spread of the disease [8].

Studies assessing the knowledge, attitudes, and practices of the public in several countries in relation to COVID-19 have either generally focused on various sociodemographic characteristics to assess perceptions, beliefs, and behaviors of the general population or focused more specifically on frontline workers like healthcare personnel [8,9,10,11,12]. A study conducted amongst young adults in Switzerland showed that men, the well-educated, wealthy people, and those without a migration background were reluctant to comply with hygiene-related practices to control the spread of the virus [13]. In Australia, the elderly and women were found to be the readiest to follow prescribed preventive measures in order to support health authorities in their effort to control the disease [14]. In Africa, physical distancing has been identified as the preventive measure with which people were most willing to comply when this was combined with incentives [15].

A cross-sectional study using a multinational sample to assess the levels and determinants of public KAP towards COVID-19 in 22 countries around the world found that the public have fair/good knowledge and practices regarding COVID-19, despite the existence of significant gaps that should be addressed [16]. Those gaps will be the focus of future awareness efforts focusing on less advantaged groups and COVID-19 preventive measure-associated negative mental health effects. The level of KAP varies from country to country. In Iran, people’s knowledge, attitudes, and practices regarding the disease are at a high level despite the existence of misconceptions about the disease [17]. Evidence from Korea shows that misconceptions surrounding COVID-19 can be avoided through the promotion of public health knowledge and belief in the efficacy of interventions by health officials and policymakers. This can lead to an increase in precautionary behaviors amongst the public [18].

Improving the KAP of the public is critical in ensuring the compliance of populations with preventive measures. Studies highlighted the need for developing effective health education programs that incorporate considerations of KAP-modifying factors [19]. Those programs can take the form of community-based health campaigns to aid in reinforcing optimistic attitudes regarding the efficacy of measures and practices for preventing spread and avoiding future outbreaks, and to avoid misconceptions surrounding COVID-19 [20]. The importance of considering cultural norms, belief systems, and perceptions in developing control measures against COVID-19 was also found to be critical [21]. A bi-national survey in Egypt and Nigeria revealed that for the public to follow standard infection prevention and control measures adequately, governments need to gain the trust of citizens by strengthening health systems and improving surveillance activities for case detection in order to offer optimum health services to their communities [22].

Studies on the compliance with prescribed measures for behavioral change after interventions during outbreaks such as those of SARS and Middle East Respiratory Syndrome coronavirus (MERS-CoV) or Ebola virus disease (EVD) have identified socioeconomic conditions as the major constraint to the adoption of recommendations [23,24,25]. For COVID-19, there are specific constraints linked to the non-compliance of populations with preventives measures in developing countries including Côte d’Ivoire: the use of overcrowded but low-cost modes of transport to travel long distances, the unavailability of public WASH facilities, limited access to clean water, crowded marketplaces, the cultural importance of social gatherings (funerals, religious worship, weddings), and lack of time for self-quarantine, especially in the case of sole providers for the family [26,27]. For a large part of the population living a hand-to-mouth existence, it is nearly impossible to respect physical distancing in daily activities, making compliance with preventive measures difficult. However, reducing the reasons for the non-compliance of populations with public health-mandated preventive measures to solely economic factors would be an oversimplification. An individual or a community exposed to health recommendations is not only a passive health care consumer. In their health-related choices, people have to navigate between competing therapeutic modalities and various types of information and health-related news, both positive and negative [28].

To address the present public health emergency, the Ivorian health authorities have prescribed the following measures: lockdown of the Grand (Greater) Abidjan conurbation (Abidjan city and its immediate surroundings), physical distancing, closures of schools and public places, curfew, closure of borders, use of face masks, regular handwashing with soap or hydro-alcoholic gel, quarantine of infected persons, bans on social gatherings, and restriction of movement. This paper aimed to assess the modalities for better compliance of the population with these preventive measures to deal with COVID-19. We hypothesized that despite the socioeconomic conditions mentioned above, the level of knowledge regarding COVID-19 and the level of trust in the government and the health system are more likely to influence health-related practices such as self-reporting/self-quarantine, physical distancing, and the use of face masks. The paper is structured around the overall objective of identifying key factors relating to non-compliance of populations in Côte d’Ivoire with preventive measures in order to tailor contextual and efficient responses and to ensure better preparedness for similar outbreaks. The argument in the paper is articulated around: (i) identifying the population’s knowledge about preventive measures regarding COVID-19 and describing the impact of COVID-19 on different populations’ lives, (ii) describing attitudes and practices related to COVID-19, and (iii) identifying hindering and fostering factors in compliance with preventive measures.

## 2. Methods

### 2.1. Ethical Consideration

The study was approved by the National Committee of Ethics of Life Sciences and Health (CNESVS) of the Ministry of Health and Public Hygiene under number 049-20/MSHP/CNESVS-kp. Furthermore, consent to participate was obtained through informed consent given online or in phone calls.

### 2.2. Study Area and Sample Size

**Figure 1:**
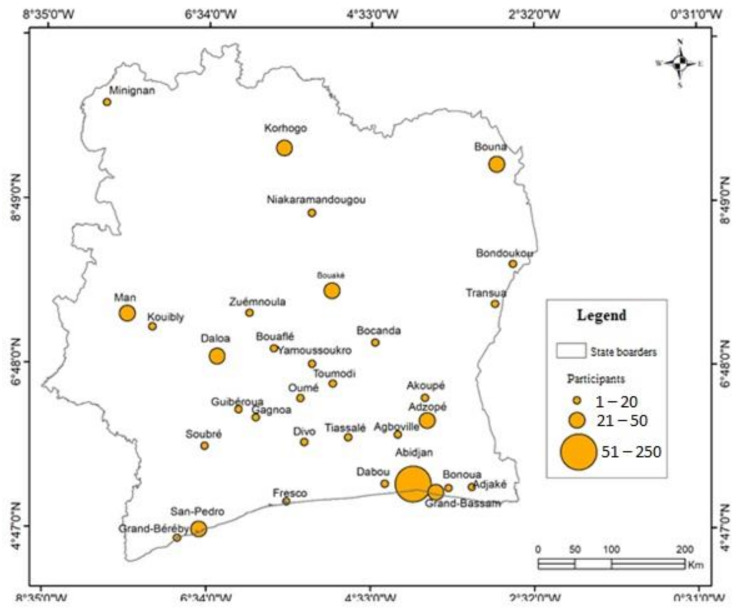
Study localities and number of participants allocated. The study was conducted from 26 April to 16 May 2020 in Côte d’Ivoire.

The study was conducted in Côte d’Ivoire. The sample size for this study n was estimated using the following formula: $n=z^{2}p\left(1-p\right)/d^{2}$, where z  is the critical value from the standard normal distribution (i.e., z = 1.96 for a confidence level of 95%) and p is the estimated proportion of the population considering the event under investigation. Here, the event is the COVID-19 pandemic (when unknown, p = 0.5), and d, the margin error (d = 0.05) [29]. Hence, a minimum size of 385 participants was expected for the study. This size was allocated according to the population size in the country’s major agglomerations and their geographical location. The sample size was allocated in proportion to the population size in these localities. In total, 32 cities and towns were identified in order to implement the study according to the above-mentioned criteria. Most of the localities were in the south and center of the country and most of the participants to be sampled were in Abidjan and its suburbs. (Figure 1).

### 2.3. Study Design and Data Collection

A multidisciplinary team representing the epidemiological, social, and medical sciences designed an online, cross-sectional survey in Côte d’Ivoire which was administered between 26 April and 16 May 2020. In order to respect the physical distance between the investigators and the respondents in compliance with preventive measures, participants were invited to complete and submit an online questionnaire in French through a Google Forms portal (https://docs.google.com/forms/u/0/ accessed on 26 April 2020). Drawing on the snowball sampling technique, each person contacted in a locality was in turn required to share the questionnaire link within his or her network. In areas where access to Internet was limited and to ensure that the required number of participants was reached, research assistants were identified in some targeted towns/villages. The role of these focal persons was to share the link of the questionnaire in their localities, provide follow up by phone calls when possible, and ensure that the questionnaires were completed. When necessary, the questionnaires were administered by phone by the focal persons for participants with no or limited access to the Internet or those with low literacy (ability to read and write in French). In a few cases, face-to-face interviews were conducted in villages while respecting physical distancing. Data collection was stopped when the time allocated for the survey was reached (i.e., three weeks).

Participants were contacted with the help of local research assistants and invited to take part in the study through social media such as WhatsApp, e-mail, and the phone.

The KAP questionnaire comprising 68 questions (Supplementary Material File S1) included topics related to the population’s perceptions of COVID-19, their knowledge about its origin, its transmission routes, respondents’ sources of information, their knowledge of preventive measures issued by the government, how they were keeping themselves safe, and their attitudes towards infected people. Lastly, the impact of the pandemic on their daily life, including on mobility and expenses, was assessed.

### 2.4. Statistical Analysis

Data collected were cleaned and analyzed using STATA version 16 (STATA Corp.; College Station, TX, USA). Only questionnaires from participants with completed data were considered for further analysis. Participants were divided into two age groups: 18 to 59 years (the young, young adults, and adults) and 60 years and above (seniors), considering employment status and education level including the less educated (those who had never been to school or had received primary level schooling) and the most educated (secondary school and university level). To compare frequency responses between groups (place of living, gender, and level of education) the chi-squared or Fisher’s exact tests were used as appropriate. Each preventive measure’s frequency index was assessed on a semi-quantitative scale stratified by the reported origin and severity of and belief in COVID-19, gender, place of residence, and education level of respondents using the Kruskal–Wallis test. Statistical significance was accepted for *p* < 0.05. The non-normally distributed pattern of the data was examined prior to data analysis.

## 3. Results

### 3.1. Sociodemographic Characteristics of Participants

Participants came from various socio-economic strata. Overall, 580 individuals were reached to participate in the survey and most of them (97.2%) consented to participate and were enrolled in and completed the questionnaire. Amongst the participants, 47.3% were females and 52.7% were males. Because one-fifth of the country’s population lives in Abidjan, over one-third of our participants were contacted in Abidjan, 39.5%, whilst 60.5% lived in other towns throughout the country. Only 6.4% were over the age of 60. Regarding the level of education, 83.0% of the participants were educated to secondary or university level, whilst 17.0% had never been to school or had stopped their schooling at primary level. Participants’ daily occupations were as follows: 9.9% were unemployed, 34.2% were full-time employees in the formal sector, 11.9% were working in the informal sector, 14.2% were self-employed, 20.4% were students, 6.9% were housewives, and 2.5% were retired.

### 3.2. Knowledge and Symptomatology of COVID-19

Table 1 depicts the knowledge surrounding the pathogen causing COVID-19 (SARS-CoV-2) according to sources of information and socio-demographic characteristics of respondents. In general, respondents had good knowledge regarding the causative agent of COVID-19. However, there were slight differences based on sociodemographic features and sources of information. About 96.3% of participants knew that a virus was the causative agent of COVID-19. This knowledge was significantly associated with living in Abidjan (99.1% vs. 94.4%; χ^2^ = 4.73; d.f = 1; *p* = 0.003), being below 60 years of age (96.8% vs. 88.9%; Fisher’s exact test, *p* = 0.038) and having reached secondary or tertiary level of education (99.6% vs. 80.2%; Fisher’s exact test, *p* < 0.001). Significant differences were found regarding the source of information of respondents’ knowledge regarding the pathological agent. Participants who had been sensitized at their place of work (98.3% vs. 93.9%; Fisher’s exact test, *p* = 0.007), place of worship (98.1% vs. 94.9%; Fisher’s exact test, *p* = 0.046), or had heard about the disease in the newspaper (98.7% vs. 93.6%; Fisher’s exact test, *p* = 0.002), in the national TV (97.2% vs. 77.8; χ^2^ = 27.07; d.f = 1; *p* < 0.001), or through social media (99.1% vs. 87.6%; Fisher’s exact test, *p* = 0.002) were significantly more likely to have good knowledge regarding the pathogen. About three-quarters of the participants had good knowledge about the length of the incubation period of the virus, but knowledge also differed according to sociodemographic features. People who had heard about the disease on television (72.8% vs. 44.4%; χ^2^ = 10.14; d.f = 1; *p* = 0.001), males (75.4% vs. 67.0%, χ^2^ = 4.84; d.f = 1; *p* = 0.028), and the most educated (75.2% vs. 53.1%; χ^2^ = 19.05; d.f = 1; *p* < 0.001) were more likely to know that this period lasts between 2 and 14 days.

A large majority of respondents were able to adequately identify the most frequently cited symptoms of COVID-19. They identified: coughing (95.4%), shortness of breath (94.7%), sneezing (71.6%), headache (65.1%), cold (63.1%), fatigue (53.6%), runny nose (42.9%), fever (39.4%), loss of taste (27.5%), perspiration (17.7%), vomiting (12.1%), diarrhea (11.7%), dizziness (7.5%), and loss of smell (1.1%) as the main signs of COVID-19. However, there was no statistical difference in their responses according to their sources of information and sociodemographic features.

### 3.3. Origin and Severity of COVID-19

The study showed that various origins of the virus were considered by the population, ranging from a simple naturally occurring disease to a man-made or even supernatural disease. Almost two-thirds of the respondents (64.8%) considered the disease origin to be the same as that of common diseases. The remaining third of respondents believed the disease had non-natural causes such as divine punishment (16.4%), genetic manipulation (12.8%), and wrath of nature (2.4%), or considered it as a mystery (2.2%). Table 2 depicts the perception of the severity of COVID-19 according to the sources of information and socio-demographic characteristics of the respondents. About 93.8% of participants considered SARS-CoV-2 infection to be a serious illness, irrespective of their perception of the origin of the virus. However, the perceived severity of the disease significantly differed according to the source of information and gender, as well as the level of education of respondents. People who informed themselves via television (94.8% vs. 74.1%; χ^2^ = 18.95; d.f = 1; *p* < 0.001), women (96.2% vs. 91.6%; χ^2^ = 5.27; d.f = 1; *p* = 0.022), and the most educated (*p* = 0.019) were likely to state that COVID-19 was a serious disease. The severity of the pandemic was explained by participants through the following reasons: its high morbidity and fatality rate (90.4%), its high contagiousness (86.4%), the unprecedentedness of the measures taken (including lockdown, wearing of face masks, and social distancing) (82.1%), and the media hype around the disease (79.3%). All these reasons led to the perception of COVID-19 as a particularly severe disease.

### 3.4. Risk Perception of COVID-19

Participants assessed both the risk of exposure and the characterization of COVID-19. In general, they believed that everyone was globally exposed to the virus regardless of gender, race, and age (92.0%). When it came to the risk of infection, respondents estimated that persons with comorbidities such as chronic disease (90.6%) and the elderly (95.0%) were most vulnerable to the disease. However, white (51.2%), and Asian people (49.7%), were perceived to be the most at risk of developing the disease as compared to black people (41.0%), and adults (50.4%) were considered to be more at risk than children (32.3%) and young people (34.8%) from all racial groups.

The respondents assessed their risk of contracting COVID-19 on a scale of “no risk” to “high risk”. Overall, we found that half of the participants did not see a great risk of themselves being infected (no risk (7.2%) and low risk (44.7%)), whereas the other half estimated themselves to be at moderate risk (27.0%) or high risk (21.1%). The factors influencing the self-risk assessment were manifold and were related to their general attitudes and perceptions, age, environment (workplace and home), adoption of preventive measures, and health status. Those who estimated that they were at low risk of being infected by COVID-19 put forward the fact that either they were young (46.2%), in good health (48.4%), washed their hands regularly (48.5%), were wearing face masks in public spaces (49.1%), and lived in safe environments (51.2%) or were self-confined (55.3%). However, those who reported being at high risk were those with disability (55.2%) and the elderly (56.1%). Roughly one-third of participants viewed their workplace as representing a low risk (35.6%) of being infected with COVID-19, whilst this risk was moderate (33.3%) for another third, and a little less than one-third found their workplace to present a high risk (27.6%).

### 3.5. Source of Information and Trust

Despite the proliferation of new sources of information and communication, the national television channel remained the primary source used by the participants. In fact, about 95.2% of the respondents learnt about COVID-19 through this channel, followed by social media (75.7%), family networks (71.3%), radio (64.2%), friends (57.8%), newspapers (52.8%), the workplace (52.8%), places of worship (45.6%), and the market (38.5%). It is particularly noteworthy that only 30.5% of respondents received information on COVID-19 directly from medical sources (the hospital).

**Figure 2:**
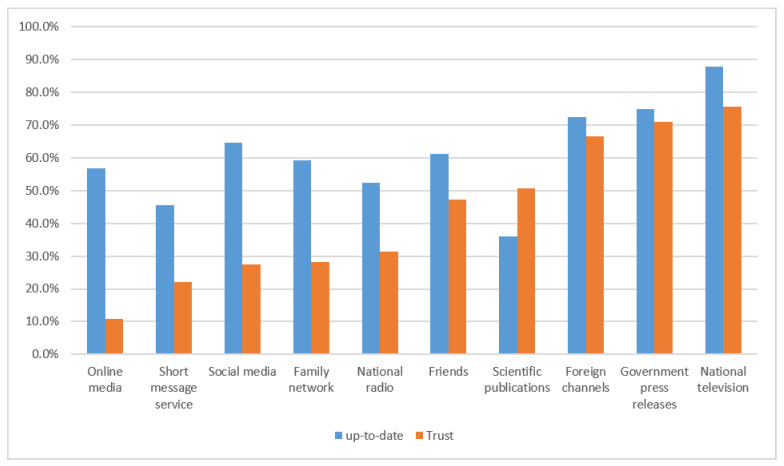
Respondents’ sources of information to stay up-to-date on the COVID-19 epidemic and their trust in these sources. The study was conducted from 26 April to 16 May 2020 in Côte d’Ivoire.

Figure 2 depicts the sources of information used by respondents to stay up-to-date on the COVID-19 pandemic and their trust in these sources. National television, government press releases, foreign broadcast channels, and social media were the main sources used by participants to keep abreast of the COVID-19 epidemic on a regular basis and in which they trusted. Although national television was amongst the main sources, its use significantly differed by gender (women: 91% vs. men: 84.5%, χ^2^ = 6.97; d.f = 1; *p* = 0.008).

Similarly, trustworthiness in this source of information significantly differed according to place of residence. Participants living in the hinterland trusted the national TV more than those living in Abidjan (82.7% vs. 65.0%, χ^2^ = 22.90; d.f = 1 *p* < 0.001). However, when considering the level of literacy of participants, educated people had less trust in national television (74.2% vs. 83.3%, χ^2^ = 3.66; d.f = 1; *p* = 0.056), but the difference was not statistically significant.

### 3.6. Trust in the Management of the Pandemic

Trust in the government regarding how the disease was managed varied, but trust in the government response was the prevailing opinion. About 35.6% trusted that the government was handling the epidemic well (9.9% very confident and 25.7% confident) and 34.6% of the respondents were moderately confident. Only 17.0% were distrustful of the way the pandemic was being managed and 12.8% did not express their opinion. Most of respondents acknowledged that the different measures prescribed by the government, including lockdown of Grand Abidjan (83.2%), physical distancing (91.1%), closures of schools and public places (92.0%), curfew (92.7%), border closures (93.8%), use of face masks (94.9%), quarantine of infected persons (94.9%), and free care for COVID-19 patients (96.1%), were likely to significantly limit the spread of the virus.

However, the evaluation of the effectiveness of some of these measures differed according to sociodemographic characteristics. Regarding the lockdown of Grand Abidjan, a significant difference in its perceived effectiveness was found regarding the place of residence. Respondents living in the hinterland found it more effective compared with those living in Abidjan (94.9% vs. 81.6%; χ^2^ = 23.65; d.f = 1; *p* < 0.001). This was also found for women as compared to men (92.7% vs. 86.7%; χ^2^ = 4.86; d.f = 1; *p* = 0.028). A significant difference in judging physical distancing effective was only found between people living in the hinterland as compared to those living in Abidjan (96.9% vs. 92.0%; χ^2^ = 5.14; d.f = 1; *p* = 0.023). Regarding the closure of schools, a significant difference regarding its perceived effectiveness was also found. People aged from 18 to 59 years old found it effective compared to those aged 60 years old and above (94.1% vs. 81.8%; χ^2^ = 7.49; d.f = 1; *p* = 0.006). The closure of public spaces was significantly found to be effective by people living in the hinterland as compared to those living in Abidjan (97.5% vs. 92.8%; χ^2^ = 7.02; d.f = 1; *p* = 0.008). This was also found for women as compared to men (98.8 vs. 92.7%. χ^2^ = 12.10; d.f = 1; *p* = 0.001) and people aged from 18 to 59 years old as compared to those aged 60 years old and above (96.3% vs. 85.7%; χ^2^ = 8.62; d.f = 1; *p* = 0.003). Regarding the curfew, its effectiveness was significantly acknowledged by respondents living in the hinterland, where its implementation was less restrictive, as compared to those living in Abidjan (80.2 vs. 72.1; χ^2^ = 5.42; d.f = 1; *p* = 0.002). This was also found for women as compared to men (81.9% vs. 73.2%; χ^2^ = 5.70; d.f = 1; *p* = 0.017) and the highly educated as compared to less-educated people (79.3 vs. 67.1%; χ^2^ = 5.87; d.f = 1; *p* = 0.015). Regarding the closure of borders, highly educated people significantly acknowledged such measures as effective as compared to those less educated (95.5% vs. 85.4%; χ^2^ = 13.95; d.f = 1; *p* < 0.001). The quarantine of people with COVID-19 symptoms was more often judged as effective by well-educated compared to the less educated (98.9% vs. 94.1, χ^2^ = 9.43; d.f = 1; *p* = 0.002).

### 3.7. Impact of COVID-19 on the Lives of the Population

Generally, the COVID-19 pandemic has had a negative impact on respondents’ livelihoods. Amongst the respondents of this survey, 4.1% had lost their jobs and 36.2% had partially stopped working. Regarding job sectors, about 0.5% of the fully employed, 3.0% of workers from the informal sector, and 11.2% of the self-employed claimed to have lost their jobs. Meanwhile, 26.9%, 41.8%, and 50% of respondents with the mentioned employment statuses had partially stopped working, respectively. Respondents felt that the following restrictive measures had negatively impacted their income-generating activities: closure of worship places (60.6%) the restriction of movement (43.6%), curfew (40.6%), the lockdown of Grand Abidjan (38.5%) and the closure of restaurants (31.0%). Regarding the cost of living, about 70% of the respondents said that their daily expenses had increased and that they lacked money for their needs. The preventive measures that were issued to mitigate the spread of the virus have impaired the population’s social lives, particularly curfew and social distancing. In fact, 87.1% of respondents stated that they could no longer attend worship and spiritual meetings nor attend social events like parties (61.9%) or funerals (64.9%) and visit close friends and relatives (79.4%). In consideration of this negative impact, more than 40% of participants found themselves in a state of anxiety and stress.

Nonetheless, there was a positive impact on people’s life that needs to be considered. In fact, more than 80% of respondents recognized that they were now paying more attention to their hygiene and lifestyle, more than half (56.6%) said they had more time to interact with their family members, and about half of the respondent (52.1%) said they had more time to chat with friends.

### 3.8. Knowledge Regarding Preventive and Protection Measures

About 99.5% of the respondents had good knowledge regarding the preventive measures prescribed by authorities to contain the spread of the virus. Those measures were: curfew, the partial lockdown of Abidjan and its immediate surroundings (Grand Abidjan), the closure of schools and universities, physical distancing, the closure of leisure and gathering areas, border closures, face mask-wearing, quarantine of suspected cases, and free care for infected people. Though these measures were well known, their appreciation varied. In general, all these measures were praised by all participants, except for the curfew, which caused a great deal of stress to the population.

Regarding the management of COVID-19 cases, almost 95.9% of the participants indicated that the hospital was the place to go for better care. The response depended significantly on the source of information, the place of sensitization, age, and level of education. Indeed, people sensitized at hospital (98.80% vs. 94.6%; χ^2^ = 12.88; d.f = 2; *p* = 0.020), who had obtained information from social media (98.1% vs. 89.1%; χ^2^ = 25.96; d.f = 2; *p* < 0.001), were aged below 60 years (96.6% vs. 86.1% χ^2^ = 9.55; d.f = 2; *p* = 0.012), and who were well educated (97.0% vs. 90.6%; χ^2^ = 12.88; d.f = 2; *p* = 0.004) were those who said they would go to hospital for treatment in the case of symptoms. When it came to the best attitudes that respondents should have on being exposed to the virus, approximately 98.8% of participants were willing either to go to hospital or to self-quarantine and call the emergency services. Respondents who obtained information from the radio (100% vs. 96.5; Fisher’s exact; *p* = 0.001) and social media (99.5% vs. 96.4%; Fisher’s exact *p* = 0.003) were likely to act in this manner.

### 3.9. Attitude towards Infected People and Self-Reported Adoption of Preventive Measures

Table 3 details the attitudes towards people with symptoms of COVID-19 according to sources of information and socio-demographic characteristics of respondents. In total, 69% of participants stated that if they were confronted with a person suspected to have the virus they would protect themselves and assist that person, i.e., call an emergency number. We found significant differences in the adoption of preventative measures according to the place and sensitization means, place of residence, age, and education. Respondents sensitized at their place of work (74.5% vs. 62.8%; χ^2^ = 9.01; d.f = 1; *p* = 0.003), at the hospital (79.1% vs. 64.5%; χ^2^ = 11.79; d.f = 1; *p* = 0.001), in newspaper (73.5% vs. 63.9%; χ^2^ = 6.03, d.f = 1; *p* = 0.014), at their place of worship (75.1% vs. 63.8%; χ^2^ = 8.28; d.f = 1; *p* = 0.004), or through television (70.4% vs. 40.7%; χ^2^ = 10.56; d.f = 1; *p* = 0.001), or social media (72.2% vs. 49.6%; χ^2^ = 31.62; d.f = 1; *p* < 0.001) were likely to protect themselves, and assist a person presenting COVID-19 symptoms as compared to other informants. Individuals living in Abidjan were more likely to respond that way compared to those living in the hinterland (73.8% vs. 64.5%; χ^2^ = 8.00, d.f = 1; *p* = 0.005), as were individuals less than 60 years old (70.6% vs. 44.4%; χ^2^ = 10.81; d.f = 1; *p* = 0.001), and the most educated (73.9% vs. 44.8%; χ^2^ = 31.61, d.f = 1; *p* < 0.001).

**Figure 3:**
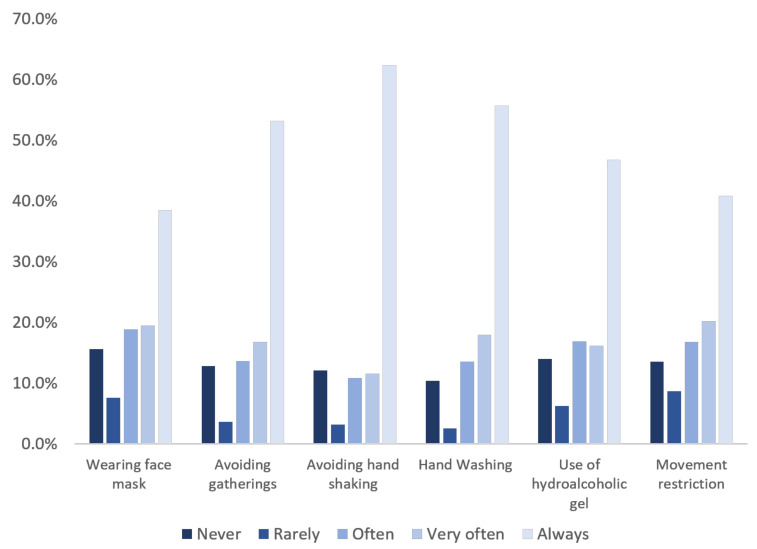
Adoption of issued preventive measures for the management of the COVID-19 by the respondents. Study conducted from 26 April to 16 May 2020 in Côte d’Ivoire.

Despite the respondents’ demonstrated knowledge about and attitude towards COVID-19, they did not fully abide by all preventive measures issued by health authorities to control the disease. Figure 3 details respondents’ self-reported behavior with regard to preventive measures. In fact, only 38.5%, and 40.8% always wore face masks when in public places (the measure not being mandatory at the time of the survey) and had limited their mobility, respectively. When travelling, 45.9% of respondents stated that they wore a face mask often or very often, whilst 15.6% never wore this item. Only 13.5% of respondents stated that they always wore a face mask when travelling (mask-wearing on public transport being mandatory). The most adopted preventive measures were avoiding gatherings (53.2%), avoiding hand shaking (62.4%), and practicing regular handwashing with soap (55.7%).

Several reasons were given by respondents regarding their attitudes to the use of face masks and the practice of regular handwashing. Regarding face masks, 11.2% of individuals did not know where to buy this item at the time of the survey. Over one-third of respondents could not afford them because they found them expensive. Some individuals (41.1%) were not using face masks either because they forgot (41.1%) or because it was uncomfortable (49.7%). Almost all respondents (97.4%) acknowledged the benefit of regular handwashing to prevent disease transmission; however, their attitude varied when it came to practice. The most important obstacles to handwashing were negligence and forgetfulness (48.8%). Few participants did not fully abide with that measure either because they did not perceive any risks that justified frequent handwashing (6%), because they always felt clean (14.4%), or they did not see the need to do so out of habit (22.3%). For others, the reasons were either that water was not always accessible (17.2%) or because soap was expensive (9.7%). Regarding the restriction of movement, respondents felt unable to abide by it because they needed to leave home for professional reasons (61.5%), for shopping (74.5%), or to seek healthcare from the hospital (51.1%). Some went out for visits and parties with friends (19.0%), for physical exercise (24.3%), and for visits to family members (28.4%).

Compliance with the preventive measures depended mainly on people’s perceptions of the disease and socio-demographic factors. Table 4 summarizes the frequency index of preventive measures assessed on a semi-quantitative scale. Regarding the origin of COVID-19, participants who argued the disease was caused by a pathogenic agent with a simple medical explanation were likely to comply with wearing a face mask (3.4 vs. 2.8 and 2.9; χ^2^ = 12.99; d.f = 4; *p* = 0.013), avoiding gatherings (3.7 vs. 2.2 and 2.3; χ^2^ = 10.23; d.f = 4; *p* = 0.033), avoiding handshaking (3.9 vs. 3.2 and 3.3; χ^2^ = 13.57; d.f = 4; *p* = 0.019); washing hands with soap (3.9 vs. 3.3 and 3.5; χ^2^ = 10.82; d.f = 4; *p* = 0.022), using hydro-alcoholic gel (3.5 vs. 2.8 and 32; χ^2^ = 15.00; d.f = 4; *p* = 0.006), and limiting movement ((3.5 vs. 2.9 and 2.9; χ^2^ = 17.61; d.f = 4; *p* = 0.001) compared to those who believed that the virus was laboratory-made or had a mystic origin such as God’s punishment or a nature’s wrath. The same trend was observed in those who stated that COVID-19 was a serious disease as compared to those who did not take it seriously with regard to wearing face masks (3.2 vs. 2.1; χ^2^ = 24.09; d.f = 4; *p <* 0.001), avoiding gatherings (3.6 vs. 2.4; χ^2^ = 21.12; d.f = 4; *p <* 0.001), avoiding hand shaking (3.8 vs. 25; χ^2^ = 13.57; d.f = 4; *p <* 0.001), washing hands with soap (3.8 vs. 28; χ^2^ = 24.78; d.f = 4; *p <* 0.001), using hydro-alcoholic gel (3.4 vs. 2.5; χ^2^ = 24.69; d.f = 4; *p <* 0.001), and limiting movement (3.4 vs. 21, χ^2^ = 26.03; d.f = 4; *p <* 0.001). Likewise, people who believed that COVID-19 was real were likely to follow the abovementioned preventive measure as compared to those who believed that the disease was fake and adhered to conspiracy theory in terms of wearing a face mask (3.2 vs. 2.2; χ^2^ = 16.64; d.f = 4; *p =* 0.002), avoiding gatherings (3.6 vs. 2.4; χ^2^ = 13.28; d.f = 4; *p =* 0.001), avoiding hand shaking (3.7 vs. 26; χ^2^ = 15.80; d.f = 4; *p =* 0.003), washing hands with soap (3.7 vs. 29; χ^2^ = 25.78; d.f = 4; *p <* 0.001), using hydro-alcoholic gel (3.4 vs. 2.6; χ^2^ = 12.24; d.f = 4; *p =* 0.016), and limiting movement (3.3 vs. 24, χ^2^ = 10.22; d.f = 4; *p =* 0.037). The same trend was observable with well-educated people with respect to wearing face masks (3.3 vs. 2.7; χ^2^ = 22.55; d.f = 4; *p <* 0.001), avoiding gatherings (3.6 vs. 32; χ^2^ = 22.01; d.f = 4; *p <* 0.001), avoiding handshaking (3.7 vs. 36; χ^2^ = 21.75; d.f = 4; *p <* 0.001), washing hands with soap (3.7 vs. 36; χ^2^ = 46.80; d.f = 4; *p <* 0.001), using hydro-alcoholic gel (3.5 vs. 2.8; χ^2^ = 47.40; d.f = 4; *p <* 0.001), and limiting movement (3.3 vs. 29, χ^2^ = 19.21; d.f = 4; *p <* 0.001) compared to the less educated. Regarding gender, the assessment of practices of respondents revealed that women were more likely to avoid handshaking (3.9 vs. 3.6; χ^2^ = 19.74; d.f = 4; *p* < 0.001) and wash their hands regularly with soap (3.8 vs. 3.7; χ^2^ = 16.49; d.f = 4; *p* = 0.002) compared to men. Regarding the use of a face mask (3.4 vs. 3.0; χ^2^ = 22.07; d.f = 4; *p* < 0.001) and use of hydro-alcoholic gel (3.4 vs. 3.3; χ^2^ = 17.25; d.f = 4; *p* = 0.002), a significant difference was found between participants in Abidjan compared to those living in the hinterland.

## 4. Discussion

This is one of the most comprehensive hybrid (online and face to face) surveys conducted so far in Côte d’Ivoire to appraise the population’s knowledge, attitudes, and practices toward preventive measures to fight the COVID-19 pandemic. It covers knowledge regarding COVID-19 and preventive measures, its impact on life, and self-reported compliance with preventive measures. Furthermore, the survey was conducted at an early stage of the pandemic (from 26 April to 16 May 2020) and covered the entire country, involving all socioeconomic strata of the population. We hypothesized that health-related practices such as self-reporting/self-quarantine, physical distancing, and compliance with preventive measures such as regular handwashing/sanitizing and the use of a face mask were influenced by the level of knowledge regarding COVID-19 and the level of trust in the government and the health system.

Our findings demonstrate a high level of awareness of COVID-19 among the participants who frequently updated themselves on the pandemic through several information channels. In Côte d’Ivoire, news about this new disease was broadcasted daily through all media, including social media and mainstream media (such as public television channels and radio). Our study showed that respondents had heard about SARS-CoV-2 primarily through national TV channels (95.2%), which represented the primary source of information for remote communities in particular, and social media (75.7%). Since its independence, Côte d’Ivoire has established a public service news channel with broadcasts covering almost the entire country, providing news in different local languages. Alongside the mainstream media, social media have emerged as an important source of information over the last decade thanks to relatively good Internet coverage (72%) and mobile phone penetration throughout the country [30].

Despite the public showing a relatively high level of knowledge related to COVID-19, many misconceptions likely to hamper compliance with preventive measures persist in Côte d’Ivoire. Respondents were aware of the disease’s symptoms as well as the preventive measures issued for its control. In general, increased knowledge surrounding the disease should lead to a willingness to follow public health recommendations [31]. In the current study, despite this high level of knowledge about the disease and how to prevent it, respondents did not fully abide by preventive measures such as face mask-wearing, handwashing, and observing curfew. We found that compliance with preventive measures greatly depended on the individual’s perceptions regarding their likelihood of getting infected rather than the severity of the disease itself and their understanding of the transmission of COVID-19. This result corroborates the findings from Wise et al. in 2020 in the US population [32]. For example, some people in our study believed that black Africans were less vulnerable to COVID-19 compared to other racial groups. This perception of low vulnerability of black people to the virus is at odds with the scientific evidence that we have so far. Evidence from the United States has shown that black people are more affected by the pandemic compared with white Americans [33], and are at higher risk of transmission and adverse related outcomes including death [34]. Even though ethnicity and race are not often included in scientific research in the country, contributing factors to COVID-19 susceptibility among black people (or African Americans) in the United States include lower socioeconomic status, crowded living conditions, residence in densely populated parts of cities, and reduced access to hygiene products and personal protective equipment. Unemployment, or employment in more virus-exposed sectors of the job market are other related factors, as are cultural habits that hinder medical advice seeking [35]. In Côte d’Ivoire, about one-third of respondents attributed the origin of the virus to a non-natural origin (man-made or supernatural). This non-natural cause of disease rhetoric is either part of conspiracy theories surrounding the outbreak or is related to cultural etiologies usually used to explain sudden outbreaks of diseases such as Ebola in Africa [36]. In those contexts, whilst global epidemic response efforts are framed by the biomedical understanding of the disease, there are diverse local meanings embedded in experiential knowledge and socio-cultural logic [37]. People who believed that the COVID-19 was a disease caused by a pathogenic agent with a simple biomedical explanation were eager to comply with the preventive measures. Furthermore, about one-third of participants who believed either in conspiracy theories or argued that the virus was of man-made or metaphysical origin (such as punishment from God or nature) were more reluctant to adopt the prescribed preventive measures. Misconceptions surrounding the origin of the disease and local representations are likely to hamper the compliance of the public with preventive measure against the pandemic. Therefore, the consideration of cultural norms, belief systems, and perceptions in developing control measures against COVID-19 are critically important [21].

Despite their capacity for spreading misinformation, social media have also been at the heart of dissemination of health information on the disease. From its onset in China, COVID-19 was perceived as a global threat to the world, not only to public health, but also to socio-economic sectors for all countries. In the absence of any effective control measures against this new virus, the role of the media has been instrumental. They have been in the frontline in raising awareness regarding the signs of the disease and preventive measures and mitigation in order to limit the spread of the virus [38]. Whilst the national media remains the primary source of information, the level of trust in the delivered information was low, especially among people living in Abidjan, men, and people with a high level of education. The image of the national news channel suffered during the political crises that Côte d’Ivoire experienced from 2002 to 2011. During this period, the national TV channel came to be perceived as a propaganda tool in the hands of the ruling party [39]. As a result, many people tend to politicize public health information. This trend was observed in the United States during the first days of the pandemic. Democrats were prompt to comply with preventive measures like the use of masks and avoid gatherings as compared with Republicans [31]. In the current study, people were found to trust and believe in information emanating from official national channels rather than social media. The COVID-19 outbreak has generated an “information tsunami” [40], and social media have been instrumental in rapid knowledge dissemination by spreading new and important information, sharing information on diagnostics, treatment, and follow-up protocols, and comparing different approaches from other parts of the world. However, they have also contributed to information pollution through possible dissemination of fake data, myths, and pessimistic information [40]. The spread of fake news or the ‘infodemic’ through rumors or false information could represent a risk in the management of a health crisis of this kind [41]. However, despite their drawbacks, Reuben et al. [20] found that social media and the Internet contributed significantly to the acquisition of needed knowledge on COVID-19 in Nigeria. It is therefore up to each user to verify the veracity of the information circulating on the web through social networks.

Despite the threat of the ‘infodemic’, our study showed that most respondents were aware of the causative agent of the disease as well as its associated symptoms. This could be interpreted as being due to the success of awareness campaigns. Apart from media, other sites of social interaction, such as workplaces and places of worship, seem to have played an important role in the acquisition of this knowledge [30,42]. However, this knowledge was not uniform among the respondents and varied according to their socio-demographic characteristics. For example, people aged 60 years and above, the less educated, and those living in the hinterland had poor knowledge of the disease. In Côte d’Ivoire, the retirement age for workers is 60. Therefore, this social category is less likely to be reached by awareness campaigns targeting the workplace. Abidjan is a large city that concentrates almost all the country’s essential services (including good Internet coverage); thus, it is important to consider that there social media were more frequently used as a source of information, alongside conventional media such as national and foreign television channels. New platforms of communication like WhatsApp and Facebook, to highlight just a few, offer users new possibilities in order to create, access, and share information with each other in a timely manner. This asymmetric access to good information between large cities and the hinterland in developing countries constitutes a threat to the management of the pandemic and may facilitate the spread of fake news wherever robust awareness mechanisms are lacking.

The misconceptions of the etiology of the disease had a negative effect on the compliance with preventive measures to control the spread of the virus. These kinds of beliefs and behaviors, if not addressed, could prevent the control of the pandemic, and undermine the response plan implemented so far. In our study, only people who considered that the disease was due to an infection caused by a pathogenic agent were quick to comply with the preventive measures. In addition, compliance with preventive measures in this study was influenced by the participant’s perception of the disease regarding its severity. This highlights the importance of access to reliable basic information in communities to avoid risky behaviors that could endanger well-being [43]. This access to reliable information is possible through the development of effective health education programs incorporating considerations of KAP-modifying factors [19]. Among the underprivileged and vulnerable population groups, the dissemination of health education via indigenous languages should be intensified [20]. Furthermore, in the context of lack of communication and knowledge around recovered patients, awareness campaigns should contribute to the reduction of misconceptions and stigma surrounding these individuals in the community [17]. Despite poor socioeconomic conditions, the level of knowledge related to COVID-19 and the trust of people in the government and the health system are more likely to influence health-related practices such as self-reporting, self-quarantine, physical distancing, and the use of a face mask.

As has been hypothesized in this study, one of the major prerequisites for compliance with preventive measures is trust in the government and health system. In the Ivorian context, as in most low-income countries where economies are fragile and still informal and the health system is very weak, restriction measures have crippled the local economy. Experience with Ebola suggests that in most African settings, centralized state control is less likely to be effective and more likely to generate mistrust [37]. In Côte d’Ivoire, the volatility of the fragile socio-political context, which has resulted in the multi-polarization of political leadership (especially prior to presidential elections in the country), has contributed towards shaping a sentiment of distrust among certain segments of the population regarding ruling political leaders, the government, the national health system, and health information and statistics emanating from governmental sources or national media. Some people believe that the government is downplaying the actual situation related to the COVID-19 epidemic in the country. Consequently, the level of trust in the management of the disease by health authorities was not found to be optimal. Irrespective of the context and continent, determinants of successful responses have been identified as being state capacity, social trust, and leadership. Countries that can successfully combine “a competent state apparatus, a government that citizens trust and listen to, and effective leaders” are more likely to perform better in their response [44]. In a KAP study on Egypt and Nigeria that was also conducted in April and May 2020, only 22.0% of the respondents expressed satisfaction in their governments’ management of the crisis [22]. The level of complete trust (35.0%) and moderate trust (35.0%) expressed in Ivorian institutions was comparatively high. Threats from COVID-19 are not considered only as a public health issue; at a certain scale the health crisis can become a security issue, and thus, a potential source of political instability [45]. Most African governments have understood this and have taken stringent measures compromising freedom, contributing towards anger towards the state due to the perceived “unpopular decisions” [46,47]. They need to gain the trust of citizens by strengthening their health systems and improving surveillance activities for detecting cases in order to offer optimum health services to their communities [22].

Another determining factor in improving compliance was linked to the practicality of preventive measures. Indeed, in this study, the restriction of gatherings and handshaking were the preventive measures with which people complied with the most. At the time of the survey, law enforcement regarding gatherings was very tough. The security forces enforced it and any offender was reported and punished according to the law. Avoiding handshakes is a well-known practice that has already been engrained into the habits of the population in Côte d’Ivoire, since it was one of the preventive measures prescribed during the 2014–2016 Ebola disease outbreak in West Africa. Compliance of the population with the different prescribed preventive measures enabled the country to prevent the spread of the virus in its territory. Regarding the use of face masks in public, people did not totally abide by that measure because of its discomfort, its inaccessibility at the beginning of the pandemic, or due to forgetfulness. One should note that people in Africa are not as accustomed to its use as compared to the populations of Asian countries such as China, South Korea, and Japan, where masks are used to reduce the risk of respiratory infection due to air pollution and air-borne pathogens [48,49]. Nevertheless, the trade in face masks and the mandatory use of masks on public transport and in public places has helped to increase their use. The instrumental importance of masks in reducing the risk of community transmission of the virus, alongside other measures like social distancing and good hygiene practices, has been demonstrated [50]. Another important aspect determining the non-compliance with preventive measures in the community was livelihood. The need to work, to feed oneself, and use health services were among several reasons why people did not comply with movement restrictions imposed to limit the spread of COVID-19. Indeed, most people in Côte d’Ivoire work in the informal sector to earn their daily living and do not have any savings to rely upon. Without a distribution plan for food, most had to continue going to the crowded markets regardless of restrictions and curfew. These findings advocate the adaptation of health messages to the different strata of the population.

Our study has several shortfalls due to the conditions of partial lockdown and restriction of movement during which data collection was conducted. First, there might be some biases due to the recruitment strategy of respondents based on the contact networks of the main investigators of the project, which resulted in more participants with a high level of education. The study faced the methodological challenges of major online surveys: the populations to which questionnaires are distributed cannot be well described and characterized, the self-selection of some respondents introduces biases in the sample, the maintenance of confidentiality is problematic, and ethical issues are difficult to address [51,52]. The second limitation was related to Internet connectivity; there was limited access in some social strata and difficulties for some informants in the completion of the questionnaire.

## 5. Conclusions

Our study showed that people were aware of the ongoing COVID-19 pandemic and the preventive measures reported in the media. Though they acknowledged these measures, opinions on how they could prevent the spread of the virus varied in the Ivorian context. The study also showed that compliance with preventive measures was rather related to people’s perceptions and beliefs about COVID-19, the nature of preventive measures, and their need to maintain their livelihoods. Our study shows that a good level of education and being of younger age correlated with a better understanding of the disease and preventive measures. Washing hands regularly with soap and avoiding gathering and handshaking were the preventive measures that individuals were most likely to comply with. In a nutshell, our findings revealed that beyond unfavorable socioeconomic conditions, the level of knowledge on COVID-19 and trust of people in the government and the health system are factors that are more likely to influence compliance with preventive measures such as physical distancing and the use of face masks, and this is consequently likely to influence the eventual acceptability and uptake of vaccines.

If individual perceptions of the pandemic are not addressed properly, the country will experience a longer health crisis. To limit the spread of the virus and increase compliance with preventive measures, we suggest developing a communication strategy to deconstruct people’s prejudices on the disease, particularly regarding its origin and the fact that all of humankind, regardless of color, race, religion, political group, level of wealth, and place of residence, are exposed to the same risk of COVID-19 infection.

## Figures and Tables

**Table 1:** Knowledge regarding the COVID-19 pathogen according to sources of information and socio-demographic characteristics of the respondents.

Source of Information	Knowledge Regarding the COVID-19 Pathogen		
	No (%)	Yes (%)	*p*
Total	21 (3.7)	543 (96.3)	
Market	335 (96.5)	208 (95.9)	0.674
Workplace	250 (93.9)	293 (98.3)	**0.007**
Family	154 (95.1)	389 (96.8)	0.333
Hospital	376 (95.9)	167 (97.1)	0.632
Newspaper	249 (93.6)	294 (98.7)	**0.002**
Place of worship	291 (94.9)	252 (98.1)	**0.046**
Another person	231 (97.1)	312 (95.7)	0.502
Radio	194 (96.0)	349 (96.4)	0.824
Television	21 (77.8)	522 (97.2)	**<0.001**
Social media	120 (87.6)	423 (99.1)	**0.002**
Sex			
Males	10 (3.4)	287 (96.6)	0.637
Females	11 (4.1)	256 (95.9)	
Place of residence			
Abidjan	2 (0.9)	221 (99.1)	**0.003**
Hinterland	19 (5.6)	322 (94.4)	
Age (years)			
18–59	17 (3.2)	511 (96.8)	**0.038**
60+	4 (11.1)	32 (88.9)	
Level of education			
None or primary school level	19 (19.8)	77 (80.2)	**<0.001**
Secondary or tertiary level	2 (0.4)	466 (99.6)	

Data were obtained from a study conducted from 26 April to 16 May 2020. Statistically significant (<0.05) differences calculated according to chi-squared (χ^2^) or Fisher’s exact tests as appropriate are highlighted in bold. No = COVID-19 is not caused by a virus, Yes = COVID-19 is caused by a virus.

**Table 2:** Perception of the severity of the COVID-19 according to sources of information and socio-demographic characteristics of the respondents.

Source of Information	Perception of the Severity of the COVID-19		
	No (%)	Yes (%)	*p*
Total	**35 (6.2)**	**529 (93.8)**	
Market	324 (93.4)	205 (94.5)	0.599
Workplace	244 (91.7)	285 (95.6)	0.055
Family	151 (93.2)	378 (94.0)	0.715
Hospital	364 (92.9)	165 (95.9)	0.164
Newspaper	249 (93.6)	280 (94.0)	0.863
Place of worship	285 (92.8)	244 (94.9)	0.301
Another person	225 (94.5)	304 (93.3)	0.532
Radio	187 (92.6)	342 (94.5)	0.370
Television	20 (74.1)	509 (94.8)	**<0.001**
Social media	126 (92.0)	403 (94.4)	0.309
Sex			
Males	25 (8.4)	272 (91.6)	**0.022**
Females	10 (3.8)	257 (96.2)	
Place of residence			
Abidjan	14 (6.3)	209 (93.7)	0.954
Hinterland	21 (6.2)	320 (93.8)	
Age (years)			
18–59	35 (6.6)	493 (93.4)	0.156
60+	0 (0.0)	36 (100.0)	
Level of education			
None or primary	11 (11.5)	85 (88.5)	**0.019**
Secondary or tertiary	24 (5.1)	444 (94.9)	

Data were obtained from a study conducted from 26 April to 16 May 2020. Statistically significant (<0.05) differences calculated according to chi-squared (χ^2^) or Fisher’s exact tests as appropriate are highlighted in bold. No = COVID-19 is not perceived as severe disease, Yes = COVID-19 is perceived as severe disease.

**Table 3:** Attitude towards a person infected with the COVID-19 according to sources of information and socio-demographic characteristics.

Source of Information	Assistance to an Infected Person: Self-Protection and Aid		
	No (%)	Yes (%)	*p*
Total	175 (31.0)	389 (69.0)	
Market	244 (70.3)	145 (66.8)	0.382
Workplace	167 (62.8)	222 (74.5)	**0.003**
Family	103 (63.6)	286 (71.1)	0.079
Hospital	253 (64.5)	136 (79.1)	**0.001**
Newspaper	170 (63.9)	219 (73.5)	**0.014**
Place of worship	196 (63.8)	193 (75.1)	**0.004**
Another person	165 (69.3)	224 (68.7)	0.876
Radio	142 (70.3)	247 (68.2)	0.611
Television	11 (40.7)	378 (70.4)	**0.001**
Social media	68 (49.6)	321 (75.2)	**<0.001**
**Sex**			
Males	89 (30.0)	208 (70.0)	0.565
Females	86 (32.2)	181 (67.8)	
**Place of residence**			
Abidjan	54 (24.2)	169 (75.8)	**0.005**
Hinterland	121 (35.5)	220 (64.5)	
**Age (years)**			
18–59	155 (29.4)	373 (70.6)	**0.001**
60+	20 (55.6)	16 (44.4)	
**Level of education**			
None or primary school level	53 (55.2)	43 (44.8)	**<0.001**
Secondary or tertiary level	122 (26.1)	346 (73.9)	

Data were obtained from a study conducted from 26 April to 16 May 2020. Statistically significant (<0.05) differences calculated according to chi-squared (χ^2^) or Fisher’s exact tests as appropriate are highlighted in bold. No = I will not assist an infected person; Yes = I will assist an infected person by protecting myself beforehand.

**Table 4:** Assessment of the compliance to preventive measures stratified according to perceptions of the origin of COVID-19 and participants’ sociodemographic features.

Perception of COVID-19 and Socio-Demographic Features with the Frequency Index of Preventive Measures	Using Face Masks		Avoiding Gatherings		Avoiding Handshaking		Handwashing with Soap		Using Hydro-Alcoholic Gel		Limiting Displacements	
	Mean	*p*-Value	Mean	*p*-Value	Mean	*p*-Value	Mean	*p*-Value	Mean	*p*-Value	Mean	*p*-Value
**Origin of COVID-19**												
Genetic	2.8	**0.013**	3.2	**0.033**	3.3	**0.019**	3.3	**0.022**	2.8	**0.006**	2.9	**0.001**
Mystical	2.9		3.3		3.4		3.5		3.2		2.9	
Normal disease	3.4		3.7		3.9		3.9		3.5		3.5	
**Severity of COVID-19**												
No	2.1	**<0.001**	2.4	**<0.001**	2.5	**<0.001**	2.8	**<0.001**	2.5	**<0.001**	2.1	**<0.001**
Yes	3.2		3.6		3.8		3.8		3.4		3.4	
**Belief in COVID-19**												
No	2.2	**0.002**	2.4	**0.001**	2.6	**0.003**	2.9	**<0.001**	2.6	**0.016**	2.4	**0.037**
Yes	3.2		3.6		3.7		3.7		3.4		3.3	
**Sex of respondents**												
Women	3.2	0.370	3.8	0.426	3.9	**<0.001**	3.8	**0.002**	3.2	0.561	3.4	0.089
Men	3.2		3.5		3.6		3.7		3.4		3.2	
**Place of living of respondents**												
Abidjan	3.4	**<0.001**	3.6	0.105	3.7	0.083	3.6	0.093	3.4	**0.002**	3.3	0.119
Hinterland	3.0		3.5		3.7		3.8		3.3		3.3	
**Level of education of respondents**												
None or primary school level	2.7	**<0.001**	3.2	**<0.001**	3.6	**<0.001**	3.6	**<0.001**	2.8	**<0.001**	2.9	**<0.001**
Secondary or tertiary level	3.3		3.6		3.7		3.7		3.5		3.3	

Data were obtained from a study conducted from 26 April to 16 May 2020. Statistically significant (<0.05) differences calculated according to the Kruskal–Wallis test are highlighted in bold. The frequency of use of preventives measures were assessed on a semi-quantitative scale (0 = never, 1 = seldom, 2 = often, 3 = very often, 4 = always) for each preventive measure.

## Data Availability

Data available on request due to restrictions with regard to privacy and ethical issues.

## Supplementary Materials

The following are available online at https://www.mdpi.com/article/10.3390/ijerph18094757/s1. File S1: Copy of Assessment of knowledge, attitudes and practices of the population and reasons for non-compliance with barrier measures in the management of COVID-19.
