# Supplementary material for: Knowledge, Attitudes, and Practices (KAP) Regarding the COVID-19 Outbreak in Côte d’Ivoire: Understanding the Non-Compliance of Populations with Non-Pharmaceutical Interventions
Source: Int J Environ Res Public Health. 2021 Apr 29;18(9):4757. doi: 10.3390/ijerph18094757 (PMC8124153; doi:10.3390/ijerph18094757)
Supplement: Supplementary file 1 [file ijerph-18-04757-s001.zip › ijerph-1150725-supplementary.pdf]

Copy of Assessment of knowledge, attitudes and practices of the population and reasons for non-compliance with barrier measures in the management of COVID-19

* Mandatory

Sans titre

INFORMATION SHEET

STUDY TITLE: Assessment of the knowledge, attitudes and practices of the population and the reasons for non-compliance with barrier measures in the management of COVID-19

IDENTITY OF RESEARCHERS AND PARTNER INSTITUTIONS This study will be conducted in collaboration with : Prof BONFOH Bassirou, Dr FOKOU Gilbert, Dr HEITZ-TOKPA Kathin - CSRS, Dr YAPI Brou Richard, Dr HOUNGBEDJI Abikpo Clarisse - CEMV-UAO, CSRS, Dr N'GUESSAN Gnagoran Kouakou Daniel - CEMV-UAO, DINDE Arlette Olaby, SANHOUN Aimé - UNA, CSRS, Dr AMIN Ariane - UFHB, CSRS, GBOKO Kossia Debia Therese (MD) - EISMV, CSRS.

OBJECTIVES OF THE STUDY

General objective

To know the factors of non-adherence of the population to the barrier measures and to determine the perception of the university community for an integrated management of the health crisis linked to COVID-19.

More specifically, it will aim to:

1. Identify the current knowledge of the target populations regarding the national measures against COVID-19;
2. Describe the attitudes and practices of the populations based on the observations of the respondents in relation to the measures enacted;
3. Identify the limiting, favourable and incentive factors for the adoption of barrier measures by the population;
4. Identify the impacts of these measures on the material, relational and subjective well-being of the population.

RESEARCH PROCEDURE

KAPB survey by questionnaire:

The aim of this study is to know and understand the practices and behaviours related to COVID-19. We have developed a questionnaire and we would like to ask you to participate. Specifically, we will ask you some questions. The main topics will be: practices and behaviours related to the measures issued by the authorities in relation to COVID-19 and their impact on your well-being.

The aim of respecting these measures is to avoid being infected and to limit the spread of COVID. Thus, having your opinions on the subject will allow us to make proposals to the decision-makers and strengthen the fight against this pandemic. It should not take more than 20-30 minutes to answer our questions.

Risks and benefits: There is no risk to you from this survey other than our intrusion into your daily activity.

Confidentiality: All information collected during this survey will be confidential. Only study members will have access to the data. We may publish key findings from this study, but your names and personal identities will not be revealed.

Consent: Participation in this study is entirely voluntary. There is absolutely no obligation to participate in this study.

Alternative participation: If you decide not to participate, this will not affect your relationship with any member of the study team in any way. Consequences if you decide to withdraw from the study and due process at the end of participation: You may decide to withdraw from this study at any time. However, we would like to make you aware that the data collected before your withdrawal may be used for reports and publications.

ETHICAL CONSIDERATION

THIS STUDY HAS RECEIVED APPROVAL FROM THE NATIONAL ETHICS COMMITTEE FOR LIFE SCIENCES AND HEALTH OF THE MINISTRY OF HEALTH AND PUBLIC HYGIENE UNDER THE REFERENCE NUMBER: N/Ref: 049-20/MSHP/CNESVS-kp

OTHER CONCERNS?

Do not hesitate to contact us if you have any other questions at the following address: Dr Yapi Brou Richard: 07995102, richard.yapi@csrs.ci
Dr Houngbedji Abikpo Clarisse : 07881283, clarisse.houngbedji@csrs.ci

Dr N'Guessan Gnagoran Kouakou Daniel: 49235995, k_daniel00@yahoo.fr Dr
Heitz-Topka Kathrin :48100854, kathrin.heitz-Tokpa@csrs.ci

1. Do you agree to participate in the study? *

Only one answer possible.

- Yes
- No *Go to question 95*

A. Identity of the participant

2. 1. Name and surname of the participant (optional)

3. 2. Place of residence *

Only one answer possible.

- Abidjan *Go to question 4*
- Hinterland *Go to question 5*

4. If Abidjan, specify the commune.

Only one answer possible.

- Abobo
- Adjamé
- Anyama
- Attécoubé
- Bingerville
- Cocody
- Koumassi
- Marcory
- Plateau
- Port-Boüet
- Songon
- Treichville
- Yopougon

Go to question 6

5. If hinterland, specify the city *

____

6. 3. Age category *

Only one answer possible.

- 18 – 29 years old
- 30 – 39 years old
- 40 – 49 years old
- 50 – 59 years old
- 60 – 69 years old
- More than 70 years old

7. 4. Gender *

Only One answer possible.

- Female
- Male

8. 5. Level of study *

Only one Answer possible.

- None
- Primary
- Secondary
- High school
- Bachelor
- Master
- Engineer
- Doctorate

9. 6. Sector of activity *

Only one answer possible.

- Employed full-time (fixed monthly salary)
- Informal/part-time employee (non-fixed monthly salary)
- Unemployed
- Housewife
- Retired
- Student
- Self-employed

10. 7. Main activity *

____

B. Current knowledge of target populations about national measures against COVID-19

11. 8. Have you heard of the coronavirus disease or COVID-19 *

Only one answer possible.

- Yes
- No

12. 9.COVID-19 is caused by *

Only one answer possible.

- a) A bacterium
- b) A virus
- c) An insect bite
- d) A fungus
- e) Other :

10. Where did you hear about the coronavirus?

13. *

Only one answer possible per line.

	Yes	No
a) On TV	◯	◯
b) On radio	◯	◯
c) In the newspapers	◯	◯
d) On the social networks	◯	◯
e) In hospital	◯	◯
f) From someone else	◯	◯
g) At the market	◯	◯
h) At work	◯	◯
i) In church / mosque	◯	◯
j) In family	◯	◯

14. k) Others, specify

11. Do you think that the coronavirus is a serious disease?

15. *

Only one answer possible.

- Yes
- No *Go to question 18*
- Don’t know

12. If yes, why do you think it is a serious disease?

16. *

Only one answer possible per line.

	Yes	No
a) it is easy to get this disease	◯	◯
b) Many people around the world suffer from it and have died from it	◯	◯
c) It is widely reported in the media	◯	◯
d) Measures have been taken that have never been taken to deal with it (containment, curfews, etc.)	◯	◯
e) Others	◯	◯

17. f) Others, specify

____
____
____
____
____

13. Which symptoms best describe the coronavirus or COVID-19 ?

18. *

Only one answer possible per line.

	Yes	No	Don’t know
a) Diarrhoea			
b) Dizziness			
c) Fever (warm body)			
d) Aches and pains			
e) Cough			
f)Vomiting			
g) Difficulties to breath			
h) Sneezes			
i) Shortness of breath			
j)Sweating			
k) Headaches			
l)Fatigue			
m) Cold / flu			
n) Runny nose			

o) Loss of taste			
p) Others			

19. q) Other symptoms, specify

14. People who can contract the coronavirus are:

20. *

Only one answer possible per line.

	Yes	No	Don’t know
a) Children			
b) Young			
c) Adults			
d) Elderly (+de 60 years old)			
e) Chinese			
f) Black			
g) White			
h) Men			
i) Women			
j) Pregnant women			
k) Everybody			
l) People suffering from chronic diseases (diabetics, hypertension, etc..)			
m) Others			

21. n) Others, specify

15. The people most vulnerable (who can easily contract the disease) to COVID-19 are :

22. *

Only one answer per line.

	Yes	No	Don’t know
a) Children			
b) Young			
c) Adults			
d) Elderly people (+de 60 years old)			
e) Chinese			
f) Black			
g) White			
h) Men			
i) Women			
j) Pregnant women			
k) Everybody			
l) People with chronic diseases (diabetic, hypertension etc..)			
m) Others			

23. n) Others, specify

How long after infection with the coronavirus does a person show signs of the disease?

24. *

Only one answer possible.

- Immediately
- 1-2 days
- 2- 14 days
- 14 - 21 days

16. The coronavirus or COVID-19 is spread by direct contact with the virus from:

25. *

Only one answer possible per line.

	Yes	No	Don’t know
a) Domestic animals			
b) Wild animals			
c) Consumption of undercooked bushmeat			
d) Infected person			
e) Air (droplets, sputum, etc)			
f) Body secretion: saliva, snot, sweat (through an object contaminated with coronavirus)			
g) Eating undercooked food (meat, eggs, etc.)			
h) Through an object soiled by the coronavirus (doors, utensils, chairs, beds, etc.)			
i) Others			

26. j) Others, specify

____

____

____

____

____

18. Can you protect yourself against the coronavirus?

27. *

Only one answer possible.

- Yes
- No *Go to question 30*

19. If yes, how can you protect yourself?

28. *

Only one possible answer per line

	Yes	No	Don't know
a) Get vaccinated	⬭	⬭	⬭
b) Wash your hands regularly	⬭	⬭	⬭
c) Wash or drink hot water	⬭	⬭	⬭
d) Wear a face mask (nose cover)	⬭	⬭	⬭
e) Avoid eating bush meat	⬭	⬭	⬭
f) Eat well-cooked food (meat, eggs)	⬭	⬭	⬭
g) Eat garlic	⬭	⬭	⬭
h) Avoid large gatherings (restaurants, places of worship, cinemas, weddings, funerals, markets, etc.)	⬭	⬭	⬭
i) Stay at home (self-containment)	⬭	⬭	⬭
j) Staying in the sun	⬭	⬭	⬭
k) Limit movement	⬭	⬭	⬭
l) Avoid touching face	⬭	⬭	⬭
m) Avoid shaking hands	⬭	⬭	⬭

n) Drinking alcohol	⬭	⬭	⬭
o) Avoid drinking alcohol	⬭	⬭	⬭
p) Avoid smoking (cigarrette)	⬭	⬭	⬭

29. q) Other attitudes to avoid covid-19, specify

____

____

____

____

____

Go to question 32

20. If no, why can't you avoid the coronavirus or COVID-19?

30. *

Only one answer possible per line.

	Yes	No
a) Spreads rapidly	⬭	⬭
b) There is no vaccine	⬭	⬭
c) Getting vaccine	⬭	⬭
d) It is a divine punishment	⬭	⬭

31. e) Other reasons, specify

____

____

____

____

____

21. Can you be cured of the coronavirus or COVID-19?

32. *

Only one answer possible.

- Yes
- No *Go to question 35*

33. 22. If yes, how can COVID-19 be cured

Only one possible answer per line.

	Yes	No	Don’t know
a) Go to the hospital			
b) Go to the pharmacy			
c) Go to the traditional healer			
d) Drinking alcohol			
e) Drinking hot water			
f)Eating garlic			
g) Taking anti-malaria drugs			
h) Taking paracetamol			
I)Do nothing (no action needed)			
j) Rest			
k) Others			

34. l) Other, specify

____

23. If you start to show symptoms and you think you have been exposed to the coronavirus or COVID-19, what would be your immediate course of action?

35. *

Only one answer possible per line.

	Yes	No
a) Isolate myself and call the COVID-19 hotline	⬭	⬭
b) Publish the news on social networks	⬭	⬭
c) Alert my employer	⬭	⬭
d) Treat it like the flu	⬭	⬭
e) Go directly to a testing centre	⬭	⬭
f) Do nothing	⬭	⬭

24. How would you rate your personal risk of contracting COVID-19?

36. *

Only one answer possible.

- Very high
- high
- Moderate
- Low
- Very low
- No risk

37. 25. What makes you think you are at the level of risk you selected in the previous question? *

Only one answer possible per line.

	Yes	No
a) I am in the young age group		
b) I have a chronic disease (diabetic, hypertension)		
c) I regularly wash my hands		
d) I smoke		
e) I drink alcohol		
f) I am self-confined		
g) I use gloves		
h) I am from a high-risk age group		
i) I am generally in good health		
j) I use a face mask		
k) My environment at work		
l) My environment at home		
m) We are all at risk		

38. Other reason(s) why you believe you are at the risk level you have selected, specify

____
____
____
____
____

26. How often do you adopt individual preventive measures?

39. *

Only one answer possible per line.

	Never	Rarely	Often	Very often	Always
a) I wear a face mask when I am outside					
b) I avoid gathering					
c) I avoid shaking hands					
d) I avoid touching my face					
e) I avoid eating badly cooked food (meat, eggs)					
f) I regularly wash my hands with soap and water					
g) I use hydroalcoholic gel					
h) I use a handkerchief for coughing and sneezing					
i) I throw the tissue in a bin immediately after use					
j) I cough into the crook of my elbow					
k) I regularly inform myself about the evolution of the disease					
l) I have quarantined myself					

m) I limit my movements					

27. Do you think that the measures taken by the health authorities are likely to effectively limit the spread of the virus?

40. *

Only one answer possible per line.

	Yes	No	Don’t know
a) Air border closure			
b) Closure of maritime borders			
c) Land border closures			
d) Distancing (1 m)			
e) Quarantine of suspected cases			
f) Management of patients			
g) Management of information			
h) Partial containment of the country (Grand Abidjan)			
i) Curfew			
j) Closure of schools and universities			
k) Mandatory wearing of face mask			
l) Closure of public spaces (bars, restaurants, maquis)			

28. How confident do you feel about the way the government is handling the response to the coronavirus threat to the country so far?

41. *

Only one answer possible.

- Very confident
- Confident
- Moderatly confident
- Not at all confident
- I have no opinion

29. Is there an advantage to washing your hands regularly?

42. *

Only one answer possible.

- Yes
- No *Go to question 44*

43. 30. If yes, why do you think there is an advantage to washing your hands regularly? *

Only one answer possible per line.

	Yes	No
a) To be always clean		
b) To avoid diseases		
c) Modern lifestyle		
d) Out of habit		
e) To be like everyone else		

31. What are the reasons why you do not wash your hands regularly?

44. *

Only one answer possible per line.

	Yes	No
a) I am always clean		
b) Out of habit		
c) By oversight / Negligence		
d) My hands do not get dirty		
e) There is no water every day		
f) Water is expensive		
g) Soap is expensive		
h) There is no place to wash my hands		
i) I use hydroalcoholic gel		
j) There is no soap		
k) There is no risk because I am resistant		

32. Do you think there is an advantage to wearing a face mask?

45. *

Only one answer possible.

- Yes
- No *Go to question 47*

46. 33. If yes, which ones? *

Only one answer possible per line.

	Yes	No
a) To avoid getting sick		
b) To avoid making others sick		
c) To do what everyone else does		
d) I don't know		

34. What are the reasons why you do not always wear a face mask?

47. *

Only one answer possible per line.

	Yes	No
a) By omission		
b) Uncomfortable		
c) I don’t know where to get them		
d) Masks are expensive		
e) I wear them all the time		

35. What are your reasons for leaving your home since the COVID-19 epidemic started?

48. *

Only one answer possible per line.

	Yes	No
a) Visiting friends	◯	◯
b) Visiting family	◯	◯
c) Going to work	◯	◯
d) Going to the market	◯	◯
e) Going to the surpemarket	◯	◯
f)Going to the pharmacy	◯	◯
g) Going to the hospital	◯	◯
h) Playing sport	◯	◯
i) Never	◯	◯

36. How many times in a week do you go out of your home since the COVID-19 epidemic started?

49. *

Only one answer possible.

- a) Every day
- b) 6 times per week
- c) 5 times per week
- d) 4 times per week
- e) 3 times per week
- f) 2 times per week
- g) 1 time per week
- h) Every two weeks
- i) 1 time per month
- j) I don't go out anymore because I am an elderly person
- k) Never

50. 37. How many hours do you estimate you have been away from home each day since the COVID-19 epidemic began? *

Only one answer possible.

- Less than 1 hour
- 1 - 3 hours
- 3 - 5 hours
- More than 5 hours
- Always at home

C. Attitudes and practices of the population in relation to the measures enacted

51. 38. What are your main sources of information about COVID-19? *

Only one answer possible per line.

	Yes	No
a) National TV channels		
b) Government (Health Ministry)		
c) National radios		
d) National newspapers		
e) Social networks (Facebook, WhattsApp, Twitter)		
f) Online information (Internet sites, Google)		
g) Foreign channels (Radios / TVs))		
h) Scientific publications		
i) Friends and acquaintances		
j) Family		
k) Email		
l) SMS		

39. What sources of information do you find reliable?

52. *

Only one answer possible per line.

	Yes	No
a) National TV channels		
b)) Government (Health Ministry)		
c) National radio		
d) National newspapers		
e) Social networks (Facebook, WhattsApp, Twitter)		
f) Official online internet sites		
g) Any website		
h) Foreign channels (Radios/TVs))		
i) Scientific publications		
j) Friends and acquaintances working in the field of health		
k) Family		
l) Email		
m) SMS		

40. How do you rate the information given by the national media on COVID-19?

53. RTI (RTI 1, RTI 2, NCI) *

Only one answer possible.

- Insufficient
- Updated
- Exaggerated
- No confidence
- I have no opinion

54. National radio *

Only one answer possible.

- Insufficient
- Updated
- Exaggerated
- No confidence
- I have no opinion

55. National presses *

Only one answer possible.

- Insufficient
- Updated
- Exaggerated
- No confidence
- I have no opinion

56. Online presses *

Only one answer possible.

- Insufficient
- Updated
- Exaggerated
- No confidence
- I have no opinion

57. Other appreciations, specify

____

____

____

____

____

41. Is COVID-19 a disease to be ashamed of?

58. *

Only one answer possible.

- Yes
- No

59. 42. How would you react to a person suspected of having or affected by COVID-19? *

Only one answer possible.

- Run away from (avoid) this person
- Assist this person
- Ashamed of this person
- Indifferent
- Make fun of this person
- Protect myself and assist
- Don’t know

43. Do you currently use medication to protect yourself from COVID-19?

60. *

Only one answer possible.

- Yes
- No *Go to question 63*

61. 44. If yes, which ones? *

Only one answer possible per line.

	Yes	No
a) Vitamin C		
b) Limon		
c) Garlic		
d) Ginger		
e) Herbal tea (tea, nime leaf, lemongrass, etc.)		
f) Parracetamol		
g) Anti-malaria medication		
h) Antibiotic		
i) Steam baths		
j) Other		

62. If other, specify

____

____

____

____

____

45. What do you think of the measures taken by the authorities for the management of COVID-19 patients?

63. *

Only one answer possible per line.

	Good	Not good	Don't have opinion
a) Construction of community diagnostic centres	◯	◯	◯
b) Setting up of free call centres	◯	◯	◯
c) Care of patients	◯	◯	◯
d) Free care	◯	◯	◯

64. 46. What other measures should be taken for the management of COVID-19 patients?

D. Limiting, enabling and motivating factors for the adoption of barrier measures by the population

65. 47. According to you, what is the origin of COVID-19? *

Only one answer possible.

- A punishment from God
- A mystical disease
- Anger of nature
- A disease like any other disease
- Other

66. 48. If other, specify

49. What means of travel do you currently use?

67. *

Only one answer possible per line.

	Yes	No
a) By feet	⬭	⬭
b) Local taxi (woro woro)	⬭	⬭
c) Taxi	⬭	⬭
d) Bus and boat-bus	⬭	⬭
e) Local bus (Gbaka)	⬭	⬭
f) Motorcycle taxii	⬭	⬭
g) Personal motorcycle	⬭	⬭
h) Personal car	⬭	⬭
i) Personal transport vehicle	⬭	⬭
j) Carpooling	⬭	⬭
k) Bike	⬭	⬭
l) Tricycle	⬭	⬭
m) Interurban transport (Massa)	⬭	⬭
n) Other	⬭	⬭

68. o) If other, specify

69. 50. Do you think someone in your family could be in a quarantine situation? *

Only one answer possible.

- yes
- No
- I don’t know

70. 51. If someone in your family should be quarantined, is there a space in your home where this person could be isolated? *

Only one answer possible.

- Yes
- No

71. 52. If there are children at home who need to be quarantined, would you be able to isolate them from the family? *

Only one answer possible.

- Yes
- No

72. 53. If there are elderly people at home who need to be quarantined, would you be able to isolate them from the family? *

Only one answer possible.

- Yes
- No

73. 54. Which of the following best describes your home? *

Only one answer possible.

- Independent house
- Flat in a building
- Common courtyard
- Studio apartment
- Informal housing/ barracks

E. Impacts of barrier measures on people's material, relational and subjective well-being

74. 55. What has changed in your employment situation because of the coronavirus? *

Only one answer possible.

- Nothing, I continue to work normally
- Not much, I have adjusted hours (minimum service) / or I work remotely (telework)
- Partial stoppage
- Dismissal / Total stoppage
- No opinion

75. 56. What has mainly changed in your habits because of the coronavirus? *

Only one answer possible per line.

	Yes	No
a) I don't have access to enough food		
b) I eat a lot more		
c) I have less money		
d) I have more money		
e) I spend more		
f) I spend less		
g) I am close to my familly		
h) I am less close to my family		
i) I pay more attention to my health		
k) I am less close to my friends		
l) I am stressed, anxious, depressed		

76. n) Other habit(s) changed because of coronavirus, specify

77. 57. Please indicate if you have bought more of the following items because of the coronavirus or COVID-19? *

Only one answer possible per line.

	Yes	No
a) Water to drink		
b) Rice		
c) Fish		
d) Meat		
e) Oil		
f) Toilet and personal hygiene products		
g) Cleaning product		
h) Medicines		
i) Soap		
j) Hydroalcolic gel		
k) Face Masks		
l) Gloves		
m) Baby food requirements		

78. n) Other item(s) you buy a lot more of because of the coronavirus, please specify

79. 58. Your expenses have increased or decreased because of the coronavirus or COVID-19? *

Only one answer possible.

- Decreased
- Increased
- No changes

80. 59. If your expenses have increased or decreased, how much do you estimate the difference per week because of the coronavirus or COVID-19?

60. Because of COVID-19,

81. *

Only one answer possible.

- a) My income has increased
- b) My income has decreased
- c) My income has not changed

61. What is the impact of the measures taken by the authorities on your work activities?

82. *

Only one answer possible per line.

	Positive	Négative	None
a) Closure of borders			
b) Containment of greater Abidjan			
c) Curfew			
d) Closure of places of worship			
e) Closure of bars			
f) Closure of maquis and restaurants			
g) Hands washing			
h) Wearing face masks			
i) Restriction of movement			
j) Respecting physical distance (1 metre)			

62. What is the impact of the measures taken by the authorities on your leisure activities *?

* Sport, virée nocturne, retrouvailles en amis, aller au cinéma, etc...

83. *

Only one answer possible per line.

	Positive	Negative	None
a) Closure of borders	◯	◯	◯
b) Containement of greater Abidjan	◯	◯	◯
c) Curfew	◯	◯	◯
d) Closer of places of worship	◯	◯	◯
e) Closer of bars	◯	◯	◯
f)Closure of maquis and restaurants	◯	◯	◯
g) Hands washing	◯	◯	◯
h) Wearing face masks	◯	◯	◯
i) Restriction of movement	◯	◯	◯
j) Respecting physical distance (1meter)	◯	◯	◯

84. 63. What is the negative impact of these measures on your work/activity *

Several answers possible.

- [ ] a) Difficulty in finding means of survival (earning money)
- [ ] b) Difficulty in finding/keeping a job
- [ ] c) Difficulty feeding the family
- [ ] d) Difficulty paying my bills
- [ ] e) Difficulties in carrying out field activities
- [ ] f) Other

85. g) If other, specify

____

86. 64. What is the negative impact of these measures on social life *

Several answers possible.

- [ ] a) Difficulty in going to places of recreation (beach, nightclub, etc.)
- [ ] b) Difficulty travelling to visit relative
- [ ] c) - Difficulty in organising important socio-cultural events (generation celebrations, weddings, baptisms, etc)
- [ ] d) Difficulties in organizing funerals for relatives
- [ ] e) Difficulties in going to church/temple/mosque to pray
- [ ] f) Other

87. g) If other, specify

____

88. 65. How long do you think you can respect and apply the measures / barriers? *

Only one answer possible per line.

	3 months	3-6 months	6-9 months	More 9 months	As long as the disease is still there
a) Closer of borders					
b) Containment of greater Abidjan					
c) Curfew					
d) Closer of places of worship					
e) Closer of bars					
f)) Hands washing					
g) Wearing of face masks					
h) Restriction of movement					
i)Respecting physical distance (1meter)					

89. 66. What would be the consequences if these measures lasted too long?

Several answers possible.

- [ ] a) Famine and misery in the country
- [ ] b) Increased insecurity (theft, rape, etc.)
- [ ] c) Drainage of state and company coffers
- [ ] d) Inability to pay salarie
- [ ] e) Increased unemployment
- [ ] f) Problem of supplying the country with foreign products
- [ ] g) Problem of supplying the cities with food
- [ ] h) Discontent of the population (riots, uprising)
- [ ] i) Other

90. j) If other, specify

67One minute with the President of the Republic. What message would you like to pass to the President of the Republic?

91. Mr. President... *

68. Do you believe in the existence of COVID-19?

92.

Only one answer possible.

- Yes
- No

93. If not, why?

Only one answer possible.

- a) A political plot
- b) An international lie
- c) A biological weapon to dominate the world
- d) Other,

94. e) If other, specify

Go to section 46 (Thank you for your contribution to the fight against COVID-19. Your answers have been taken into account).

For what reason(s) do you refuse to participate in this study

95.

Go to section 46 (For what reason(s) do you refuse to participate in this study).

Thank you for your contribution to the fight against COVID-19. Your answers have been taken into account

This content is neither written nor endorsed by Google.

Google Forms
